# Design of RNA hairpin modules that predictably tune translation in yeast

**DOI:** 10.1093/synbio/ysy019

**Published:** 2018-10-13

**Authors:** Tim Weenink, Jelle van der Hilst, Robert M McKiernan, Tom Ellis

**Affiliations:** 1 Centre for Synthetic Biology, Imperial College London, London SW7 2AZ, UK; 2 Department of Bioengineering, Imperial College London, London SW7 2AZ, UK; 3 Department of Life Sciences, Imperial College London, London SW7 2AZ, UK

**Keywords:** yeast, RNA secondary structure, translation, gene expression, modularity

## Abstract

Modular parts for tuning translation are prevalent in prokaryotic synthetic biology but lacking for eukaryotic synthetic biology. Working in *Saccharomyces cerevisiae* yeast, we here describe how hairpin RNA structures inserted into the 5′ untranslated region (5′UTR) of mRNAs can be used to tune expression levels by 100-fold by inhibiting translation. We determine the relationship between the calculated free energy of folding in the 5′UTR and *in vivo* protein abundance, and show that this enables rational design of hairpin libraries that give predicted expression outputs. Our approach is modular, working with different promoters and protein coding sequences, and outperforms promoter mutation as a way to predictably generate a library where a protein is induced to express at a range of different levels. With this new tool, computational RNA sequence design can be used to predictably fine-tune protein production for genes expressed in yeast.

## 1. Introduction

Altering the expression levels of genes is a crucial tool for synthetic biology and biotechnology applications (1–3), and in all organisms this is most-readily achieved by changing or regulating the promoter to modify the rate of transcription of the mRNA (4, 5). Natural systems, however, also regularly modify protein production by altering the efficiency with which an mRNA is translated (6), and in model bacteria changing the gene sequence to modify the rate of translation is a well-established method for predictably tuning the rate of protein production (1).

In bacteria, the rate of mRNA translation is tuned by altering the ribosome binding site (RBS) sequence found upstream of the AUG start codon where the ribosome is recruited to initiate translation via RNA: RNA base-pairing (7). As the efficiency of ribosome recruitment at the RBS defines the translation initiation rate of an mRNA, extensive work has determined how changes to the RBS sequence modify gene expression (8). This has led to several sequence-to-output predictive tools that use thermodynamic models of nucleic acid pairing to predict the binding efficiency of ribosomes to any given bacterial mRNA (7). These are enabled by the multiple software packages that predict nucleic acid secondary structures and determine their minimum free energy (MFE) of folding by summing the thermodynamic contributions of all base-pairing interactions (9–14).

For eukaryotic synthetic biology the development of similar tools that predictably tune mRNA translation rates by changing bases in the 5′ untranslated region (5′UTR) is complicated by the fact that translation initiation follows a different mechanism to bacteria, where only part of the ribosome, the 40S subunit, initially binds the mRNA (15). After binding the 5′ cap, it scans along the 5′UTR of the mRNA until reaching the first AUG start codon, which is usually preceded by a short A or G/C rich sequence in the upstream 10 bases known as the Kozak sequence (16). No direct RNA: RNA base pairing occurs between the ribosome and the mRNA, and so the effect of changing 5′UTR bases cannot be just determined from nucleic acid pairing estimations.

In yeast, efforts to predict base-to-expression relationships have mostly concentrated on understanding the effect of changing bases in the Kozak sequence area. A study varying the 10 bases upstream of the start codon on an mRNA determined the optimum Kozak sequence in yeast for efficient protein expression (17) and the data from that study have recently led to the first yeast translation design tool (the yUTR Calculator) which enables users to predictably change mRNA translation by up to 7-fold by altering the 10 bases upstream of the start codon (18). Another recent study assessing 500 000 random 50 base 5′UTR upstream of the His3 gene has taken this further, now yielding a predictive model for 5′UTR function that takes into account more than just the immediate Kozak sequence variation (19). As well as demonstrating the importance of avoiding AUG sequences within the 5′UTR creating upstream open reading frames (uORFs), this work also confirms the findings of previous studies that have shown that the presence of RNA secondary structures in the 5′UTR is a key effector of the rate of translation (17, 20–22). At least three genome-wide studies in *Saccharomyces cerevisiae* have confirmed a negative correlation between mRNA translation efficiency and the degree of 5′UTR secondary structure, especially around the start codon (23–25). Secondary structures present in 5′UTRs in higher eukaryotes, such as mammalian cells (26, 27) and plants (28), have also been shown to lead to a similar reduction in gene expression.

So alongside efforts to tune yeast expression via sequence changes at the Kozak sequence, there is now strong evidence that translation initiation efficiency can also be altered by removing or introducing secondary structure in the 5′UTR. To boost protein overexpression from commonly-used yeast plasmids, Crook *et al.* (29) redesigned multiple cloning sites specifically to remove structure-forming sequences appearing in 5′UTRs. In contrast, Lamping *et al.* (22) down tuned protein expression by inserting GC-rich stem-loop sequences into yeast 5′UTRs. Others have also changed protein expression by 5′UTR changes by adding secondary structures that are combined with other forms of RNA-based translational regulation or bound by specific RNA-binding proteins (30–33).

Given the abundance of evidence that RNA secondary structure upstream of the Kozak region can alter mRNA translation levels in yeast, we set out to develop a ready-to-use tool to reliably modify protein expression that does not involve changing the base sequence immediately next to the upstream promoter or downstream open reading frame (ORF). Specifically, we develop a new class of short modular part that can be inserted into yeast 5′UTRs upstream of the Kozak in order to predictably modulate gene expression in a way that can be designed at the sequence level using RNA folding modeling tools. Following the strategy established by the RBS Library Calculator tool (34), we use prediction of secondary structures of RNA hairpin folds to design sequences with degenerate bases that can be inserted into the 5′UTR to yield a library with a defined range of expression levels in a population of yeast. We show that hairpin sequences can be designed to fine-tune gene expression as desired and that the hairpin libraries are modular, working as predicted when paired with different promoters or protein coding sequences.

## 2. Materials and methods

### 2.1 Strains and media

BY4741 (MATa his3Δ1 leu2Δ0 met15Δ0 ura3Δ0) was used for all yeast transformations, using a high efficiency yeast transformation protocol (3). Standard practice in yeast genetics was followed (36). When used, IPTG was supplied at a concentration of 10 mM. For selection in *Escherichia coli*, antibiotics were added at the following concentrations: Ampicillin: 100 μg/ml, Kanamycin: 50 μg/ml, Chloramphenicol: 33 μg/ml.

### 2.2 Library cloning

The Yeast ToolKit (YTK) cloning system was used for library plasmid construction (37). NEB Turbo chemically competent *E. coli* (NEB C2984I) were used for transformation of library constructs, according to the manufacturer’s protocol. Supplementary Table S1 lists all multigene level plasmids constructed for the libraries in this study and their constituent cassette-level plasmids. Contrary to normal YTK protocol, one of the cassettes is an *in vitro* product created via PCR, rather than through a cloning step. The corresponding PCR reactions for each cassette part in these libraries are also shown in Supplementary Table S1 and primers are defined in Supplementary Table S2. For a detailed description of the cloning method we refer to the Supplementary Material and Supplementary Figure S1.

Cloned cassette-level plasmids used in the multigene assembly reactions and as template for the PCRs are listed in Supplementary Table S3, along with part plasmids used in their assemblies. Any used parts that were not defined as a standard part in the YTK kit are listed in Supplementary Table S4.

### 2.3 Flow cytometry analysis

Libraries were tested using flow cytometry performed with the Attune NxT Acoustic Focusing Cytometer (ThermoFisher Life Technologies), with accompanying 96-well plate reader. Two lasers are installed in this machine: blue 488 nm and a 561 nm yellow laser. Green fluorescence (blue laser) was detected at a voltage of 450 with a 530/30 nm filter, red fluorescence (yellow laser) at a voltage of 480 with a 620/15 nm filter, while forward and side scatter were detected at voltages of 40 and 340, respectively.

Strains were picked and grown to saturation in 700 μl YEP-Dextrose in 2 ml 96 deepwell plates (VWR 732-0585). Cultures were grown overnight in a shaking incubator (Infors HT Multitron MTP) at 800 rpm at 30°C, with breathe-easy film (Sigma Z380059) covering the plate to prevent evaporation. This plate was then diluted 500 times into a new deepwell plate containing 700 μl minimal dropout media with 2% galactose for induction. After overnight incubation of at least 12 h, the cultures were back diluted into a Costar 96 round-well flat bottom plate (VWR 3596). Dilution was 10–100-fold, depending on culture density, using the same media in a total volume of 300 μl per well. Cultures were grown for a minimum of 4 h before initiation of the measurements, to ensure logarithmic growth. For autofluorescence measurements the same incubation protocol was followed with the untransformed parent strain.

A total of 10 000 events were measured for each of the three biological repeats per sample. Only events with forward and side scatter values greater than 10^3^ were counted. Populations were tightly gated around the median of forward and side-scatter, in order to limit the effect of cell size on the measurements. Populations were subsequently gated for sufficient mRuby2 expression in strains that contained a constitutively expressed red fluorescent control. Conversely, in constructs with constitutively expressed yeast enhanced green fluorescent protein (yEGFP), populations were gated for sufficient green fluorescence. Gating and exporting was done using FlowJo 10.0.7r2. We defined normalized fluorescence as the fold increase over median auto fluorescence levels of *S. cerevisiae* yeast. Accordingly, raw fluorescence values of tested strains were divided by the median fluorescence of unmodified BY4741 cells, grown under identical conditions. Matlab 2016b was used for the visualization of the resulting histograms.

### 2.4 Library member sequence determination

Hairpin sequences of individual library members were determined through yeast colony PCR. Single colonies were resuspended in 50 μl 0.02 M NaOH with a sterile toothpick. After incubating this solution at 99°C for 10 min, a 2 μl aliquot was used as template in a 50 μl PCR reaction. Primers were used at 200 nM concentration, with TW188, TW149 and TW325 were used for the reaction and TW195 and TW457 used for sequencing. These are given in Supplementary Table S2. Isolates with DNA sequences that did not match the relevant library sequence space (i.e. mutations or sequencing errors) were discarded from the analysis. 5′UTR sequences of all isolated library members are listed in Supplementary Tables S5–S8.

### 2.5 Reverse transcription and qPCR

Total RNA was isolated from yeast using the YeaStar RNA Kit (Zymo Research R1002), according to the manufacturer’s instructions. Cultures were grown to saturation overnight and back diluted 1:100 the next morning. Cultures were then grown to an OD600 of approximately 2, to ensure logarithmic growth. In 1.5 ml of the culture was used for RNA isolation. This volume was adjusted to ensure that the same amount of biomass was used for every sample.

A total of 400 ng RNA of each of the samples was used in the reverse transcription (RT) reaction to produce cDNA that could be used for quantitative PCR (qPCR). The RT reaction was performed in a total volume of 10 μl, using the Tetro cDNA synthesis kit (Bioline BIO-65043) according to the manufacturer’s instructions. For each reaction, a negative control lacking the reverse transcriptase was included. Specific primers were used for the RT step, which are listed in Supplementary Table S2.

cDNA obtained in the RT reaction was diluted 300× and used for qPCR. Diluted cDNA of 4.6 μl was used in a total reaction volume of 10 μl. The Kapa universal qPCR 2× Mastermix Kit (KAPA Biosystems KK4601) was used according to the manufacturer’s instructions. The primers (0.2 μl per primer per reaction) for each of the screened targets are listed in Supplementary Table S2. Measurements were performed with the Eppendorf MasterCycler RealPlex qPCR thermocycler and accompanying software. The following cycling program was used: denaturation for 10 min at 95°C followed by 50 cycles of 15 s at 95°C, 1 min annealing and extension at 60°C.

Three technical replicates were performed for every biological sample. The data were analyzed using the 2^−ΔΔCT^ (also ‘dd-Ct’) method (38). TPI1 was used as a reference gene (39). The error was calculated as the standard deviation of the replicates with propagation of the error in the reference gene measurements. For each qRT-PCR experiment, two controls were included to monitor the level of DNA contamination of the cDNA and the used reagents. For every target a triplicate measurement of ddH_2_O was included. Secondly, for every target in every strain we included the RT control samples produced during the cDNA synthesis.

## 3. Results

### 3.1 Altering expression strength with 5′UTR RNA structures

**Figure 1.**
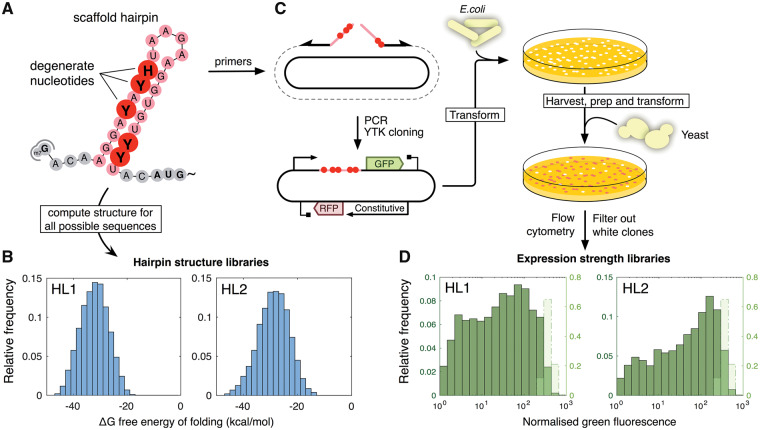
Overview of 5′UTR hairpin library HL1 and HL2 creation. (**A**) Degenerate nucleotides are inserted at various positions into the design of a hairpin scaffold. (**B**) The MFE of all possible sequences is calculated with RNAfold and visualized in a histogram. These values are then used to predict the expression profile of the resultant library. If necessary, the composition and location of the degenerate nucleotides is adjusted to produce the required distribution of expression levels. (**C**) When the design meets the requirements, primers incorporating the required degeneracies are ordered. A library of plasmids for *Escherichia coli* transformation is created using PCR and a subsequent Golden Gate based assembly step implemented in YTK format. The library of *E. coli* transformants is harvested and plasmids prepped for yeast transformation. (**D**) Transformant yeast colonies are pooled and analyzed using flow cytometry. Clones that do not show constitutive red fluorescence are discarded in quality control for correct assembly. The diversity of the library of hairpins is reflected in the spread of green fluorescence over three orders of magnitude. For reference, the distribution of the original construct is shown in light green and normalized with a secondary axis. A pre-normalization comparison is shown in Supplementary Figure S2.

Recent work in *S. cerevisiae* has shown that hairpin structures within the 5′UTR of mRNAs decrease expression by inhibiting translation (22). To first verify this finding, we used one-pot cloning methods to introduce a library of hairpin structures into the 5′UTR of an mRNA encoding expression of green fluorescent protein (GFP) from the galactose-inducible GAL1 promoter. Using a strategy of PCR amplification with degenerate oligonucleotide primers, followed by cloning with the modular YTK system, we introduced a hairpin library spaced 15 bases upstream of the start codon of GFP, as summarized in Figure 1 and detailed in Supplementary Figure S1. To design the hairpin-encoding sequences, we used RNAfold from the ViennaRNA suite of tools, as it allows the RNAfolding algorithms to run on a local computer and the thermodynamic parameters can be adjusted to work at 30°C (14, 40). As an initial test, two pairs of degenerate oligonucleotide primers were designed to create two alternative secondary structure libraries, using a design strategy that ensures no premature AUG start codons are inserted in order to avoid uORF formation. The primers together encode the introduction of 60 bases of sequence predicted by RNAfold to fold into a strong hairpin motif of a 27 basepair stem with four base loop, placed just upstream of the Kozak region (Figure 1A).

Degenerate nucleotides were designed into 10 positions within the primers so that the different constructs produced by the cloning would have variation in the bases within the hairpin stem, and therefore, a range of strengths for the resulting RNA secondary structure. Using RNAfold, the distribution of the predicted minimum free energies of folding for each of the two hairpin libraries could be determined by calculating the folding strengths for all possible combinations of introduced degenerate bases (Figure 1B). The two initial libraries, each with 10 degenerate bases were designed to give a normal distribution of predicted secondary structure strengths with average MFE of folding of −32.2 kcal/mol and −28.8 kcal/mol (libraries HL1 and HL2, respectively, see Supplementary Table S9 for sequences).

During plasmid library construction all colonies were pooled for each library, then transformed into *S. cerevisiae* and all yeast colonies from each library were then pooled. The pooled libraries were grown in galactose media to induce gene expression and the green fluorescence per cell was measured for 10 000 cells of each library by flow cytometry (Figure 1C). For both HL1 and HL2 libraries, the normalized green fluorescence was seen to vary across the population over three orders of magnitude (Figure 1D), indicating that the hairpin modules were indeed altering the expression of GFP in the cells significantly compared with an equivalent construct with no hairpin module (Supplementary Figure S2). The shape and peak of the fluorescence histograms in the two cases also differed, with more cells exhibiting low amounts of GFP expression when the library with stronger predicted secondary structure (HL1) was used.

### 3.2 Matching expression levels to predicted folding energies

**Figure 2.**
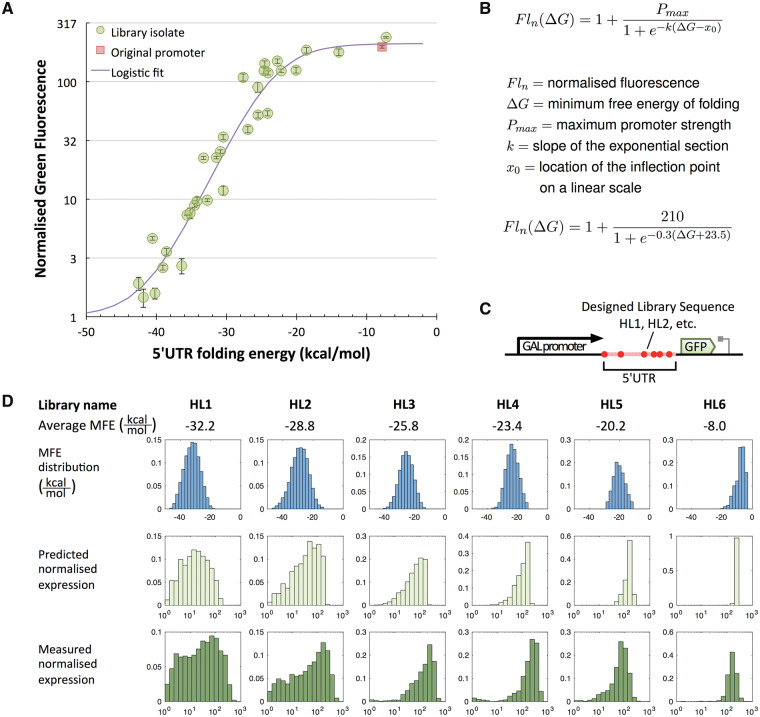
Correlation between 5′UTR hairpin strength and protein expression. Stronger hairpins are shown to cause lower expression in a predictable manner. (**A**) Determination of the transfer function between hairpin folding energy and normalized green fluorescence. Fluorescence was measured for 31 isolated HL1 and HL2 library members and divided by the median auto fluorescence of the parental strain to obtain the normalized fluorescence. The isolates were sequenced to obtain the 5′UTR sequences, which were used to calculate the corresponding hairpin folding energy. The diagram shows the folding energy of the 5′UTR of each isolate plotted against the normalized green fluorescence and fitted to a logistic growth curve. Sequences and obtained values are listed in Supplementary Table S5. Error bars indicate standard deviation of the median of three measurements of 10 000 events each. (**B**) Equation describing the logistic fit between predicted folding energy and normalized fluorescence. (**C**) Diagram of the transcription unit that constitutes the RNA hairpin library. Pink area constitutes the hairpin backbone with red spheres indicating degenerate nucleotides. (**D**) Correlation between the predicted strength of 5′UTR structure libraries and the measured gene expression distributions of these libraries. All panels show normalized frequency distributions (histograms). A total of six libraries (HL1-6) are shown, whose average MFE of folding is given in kcal/mol. In the top row of panels, the histogram of the distribution of the MFE in the 5′UTRs of the different libraries is shown. The horizontal axes for these panels ranges on a linear scale from −50 kcal/mol to 0 kcal/mol. The middle row converts these into a histogram of predicted normalized expression levels using the equation established in panel A. The third row shows the experimentally obtained distribution of normalized fluorescent reporter expression levels as measured by flow cytometry. In the lower two rows, the horizontal axis corresponds to normalized green fluorescence (a unit-less quantity) ranging on a logarithmic scale from 1 on the left to 1000 on the right.

We next sought to determine the relationship between the predicted MFE of the encoded 5′UTR secondary structures and the resulting *in vivo* GFP expression levels in *S. cerevisiae.* We randomly selected 31 individual colonies from our libraries, determined the sequence of their 5′UTR regions and used flow cytometry to characterize their GFP expression per cell upon induction. We used RNAfold to predict the MFE for the hairpin structures in each isolate by inputting their 5′UTR sequences. We then plotted the relationship between the predicted MFE and the normalized GFP expression which revealed a clear relationship between the predicted UTR folding energy and the GFP expression for each isolated colony (Figure 2A). Weak structures with MFE above −22 kcal/mol do not give a measurable decrease in expression, but as MFE values decrease further a steep decline in GFP expression is seen, in line with the hypothesis that increasingly strong secondary structures in the 5′UTR inhibit gene expression. To confirm that the decrease is due to reduced translation rather than reduced transcription, we used qPCR to verify that our transcript levels per cell remain the same, despite different 5′UTR hairpin sequences being introduced (Supplementary Figure S3).

As the trend in Figure 2A exhibits a sigmoidal curve, we chose to modify the equation for logistic growth to give an equation to predict gene expression (as normalized fluorescence) from the calculated MFE of folding of the 5′UTR sequence and the maximum output of the promoter (*Pmax*) used in the gene expression construct (Figure 2B). While this fitted curve closely follows the data, not all data points fall within the 95% confidence interval and in the most extreme cases the predicted expression values are 3-fold over or underestimated compared with the observed value. While this demonstrates that our curve fit approach doesn’t fully capture all behaviors, its predictive power still compares favorably to many existing tools. The RBS Calculator v1.0 gave deviations up to 10-fold from predicted values despite using a model derived to take into account multiple biophysical processes (8). Likewise, the linear fits in the yUTR Calculator (18) do not appear to be as close a match to data as those seen here. The fact that we constrain 5′UTR variation to just a few key nucleotides that govern hairpin strength is likely to be the reason such a simple approach performs well.

To test the predictive power of the curve fit equation, we next built and tested four further libraries (HL3-6) designed to have different average structure strengths within their distributions compared to HL1 and HL2 (Figure 2C). The predicted expression level distributions for all members of each library were determined from the calculated MFEs using the equation fitted to use the GAL1 promoter for *Pmax.* The predicted distributions were then plotted as histograms and compared with flow cytometry distributions of GFP output for each yeast library. This revealed a good qualitative agreement between the model predictions and the experimental data (Figure 2D). In all cases, the prediction histograms matched the measured normalized GFP expression in both their spread (the range of expression levels) and the position of the peak (the average expression). Some peak-broadening is seen in the flow cytometry data compared with the predictions, due to small and large cells within the population causing intrinsic noise.

**Figure 3.**
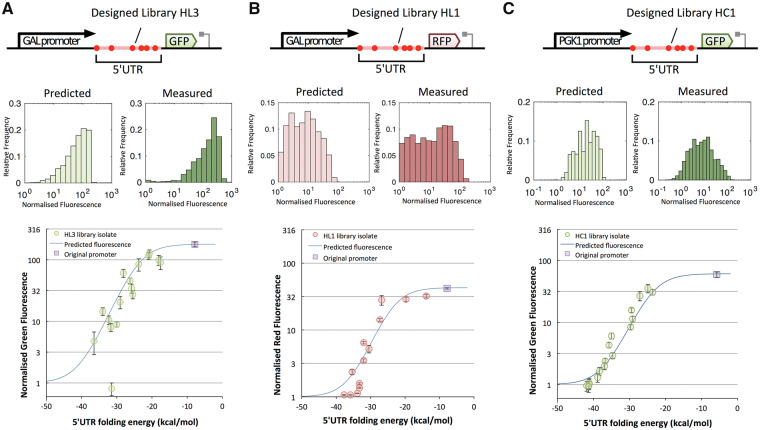
Hairpin libraries maintain predictability in different genetic contexts. Isolates from three hairpin libraries inserted into three genetic contexts show that predictability of individual sequences is maintained across the different contexts. For each genetic context, we show a cartoon of the DNA sequence (not to scale, top row), a comparison between predicted and measured distribution of normalized fluorescence (middle row), and plots of the normalized fluorescence of isolated colonies plotted against the predicted free energy of folding of the 5′UTR region as well as the predicted fluorescence at different folding energies based on the maximum fluorescence of the original promoter (bottom row). (**A**) Isolates of the HL3 library in the context of a GAL1-derived promoter and the yEGFP. The HL3 library has a mean MFE of folding of 25.8 kcal/mol, therefore, isolates with an MFE lower than −35 kcal/mol are rare. (**B**) Isolates of the HL1 library in the context of a GAL1-derived promoter and the mRuby2 RFP. Mean MFE of the HL1 library is −32.2 kcal/mol. (**C**) Isolates of the HC1 library in the context of the PGK1 promoter and yEGFP. Mean MFE of the HC1 library is −28.9 kcal/mol. Sequences and obtained fluorescence values are listed in Supplementary Tables S6–S8 for (A), (B) and (C), respectively.

To then verify that individual members in these libraries perform as expected, we isolated, sequenced and individually characterized constructs from one of these libraries (HL3) and plotted their observed GFP expression against the predicted MFE values based on their sequence (Figure 3A). Apart from one outlier, that we attribute to a GFP null mutation, an excellent agreement between prediction and measurement values is seen, verifying the ability to predictably tune gene expression by changing the sequence of the inserted hairpin.

### 3.3 5′UTR hairpins as modular parts

To demonstrate that designed hairpins can be interchanged into other constructs, we next swapped the ORF sequence in our constructs with one encoding expression of a different protein. We replaced the yEGFP ORF with the mRuby2 ORF for libraries HL4, HL2 and HL1 to create versions of these libraries that now express a red fluorescent protein (RFP). The mRuby ORF has no significant nucleotide sequence homology to the yEGFP ORF, meaning that the 5′UTR context is changed by having a different downstream RNA sequence. Flow cytometry analysis of RFP expression in these new libraries closely-matched the predicted expression distributions (Supplementary Figure S4a). Furthermore, when randomly-selected isogenic constructs were analyzed for the HL1-RFP library, we again saw a good agreement between prediction and measurement values for individual designs (Figure 3B).

We then further assessed modularity by varying the promoter sequences upstream of our GFP-encoding constructs. We first designed a 5′UTR encoding a new degenerate hairpin library (HC1) chosen to have an average MFE of −28.9 kcal/mol to ensure a wide spread of outputs with different promoters. This was then constructed into a GFP expression cassette and used to make five libraries, each with a different constitutive promoter whose expression strength had been pre-determined to obtain a known *Pmax.* Expression distributions were predicted for the HC1-based libraries using the equation fitted to the five *Pmax* values. When these predicted distributions were compared to flow cytometry analysis for all five libraries, a good qualitative match was again seen, especially for the stronger promoters (Supplementary Figure S4b). To again confirm that this was true at the individual level, isogenic constructs were sequenced and re-analyzed and these demonstrated excellent agreement between predicted and measured expression levels (Figure 3C). While this is only a small sample of possible promoters, the result implies that the 5′UTR hairpin approach is modular with respect to upstream promoters, meaning that the approach could be applied to alter the expression from many promoters in *S. cerevisiae.* It is important to note, however, that weak promoters appear especially sensitive to strong 5′UTR structure and so would be best suited to be paired with libraries with weaker average folding strengths.

### 3.4 Predictable tuning of regulated expression with 5′UTR hairpins

The approach developed here of placing designed secondary structure within the mRNA 5′UTRs offers a new modular tool to modulate gene expression. In *S. cerevisiae,* the most commonly-used method for altering gene expression strength is to replace the promoter, however, when gene regulation is required (e.g. for inducible expression) tuning at the promoter becomes problematic, as mutating bases within the promoter to change the transcription strength often also alters the efficiency with which transcription factors bind and regulate the promoter. 5′UTR hairpins offer a solution to this problem, because fine tuning of expression output is achieved by altering bases away from where the regulation is encoded.

To demonstrate this, we directly compared our approach to an equivalent regulated promoter library we produced previously using targeted mutagenesis, selection and characterization (41). In both cases a synthetically regulated version of the GAL1 promoter is used where the Lac Inhibitor (LacI) represses GFP expression by binding to its binding site placed within the promoter core. External regulation of this promoter is via the inducer IPTG which blocks the LacI repressor and so leads to full promoter expression.

**Figure 4.**
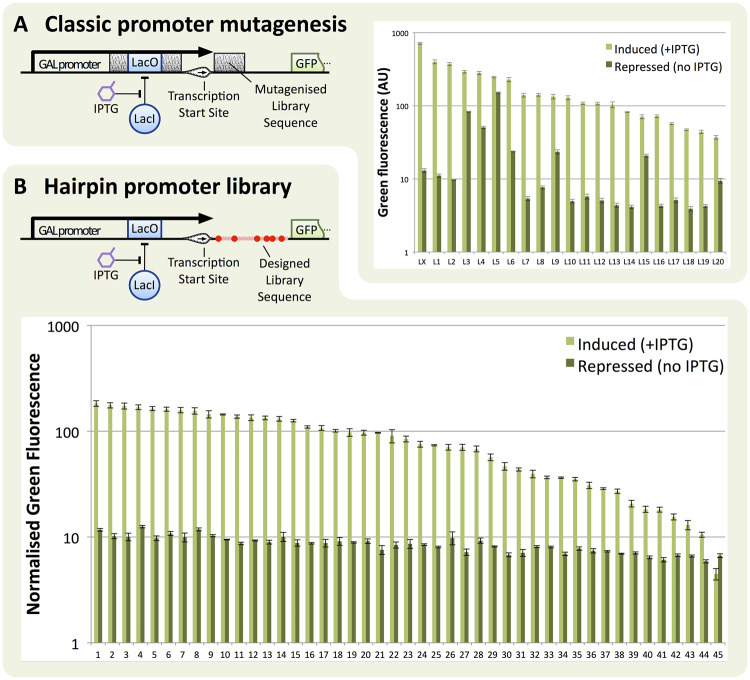
Comparison of methods for regulated expression library creation. The GAL1-based regulated promoters contain a synthetic Lac operator site which can be bound by the Lac Repressor (LacI) in order to repress the promoter. IPTG can subsequently be added to release LacI from the DNA, reversing its repressive effect and inducing yEGFP expression. (**A**) Results and method employing targeted random mutagenesis. In this approach, selected regions in the core promoter (grey blocks) are completely randomized. From a pool of 350 candidates, the 20 best performing hits (L1–L20) and the non-mutated version (LX) are selected. Error bars represent standard error of the mean of three biological repeats. (**B**) Results and method employing 5′UTR hairpins as developed in this work. A hairpin library sequence (HG1) containing degenerate nucleotides is placed directly following the transcription start site and preceding the start codon. From the resulting clones, 45 are directly picked and characterized, without a prior screening step. Error bars represent standard deviation of the median of three biological repeats.

For both libraries, a desirable range of maximum outputs is seen when IPTG is applied, but the efficiency of repression varies considerably for the promoter library, with several promoters being especially leaky (Figure 4A). In contrast, all members from the 5′UTR hairpin library show the desired expression characteristics, with repression unaffected and induced output matching predictions (Figure 4B). Furthermore, generation of this 45-member library was performed in under a week with only three colonies from 48 needing to be discarded due to no expression. In contrast, the promoter mutagenesis method used previously required 2–3 weeks and the screening of over 300 colonies to isolate the 21-member graded library (41).

## 4. Discussion

The work described here offers a new modular tool for yeast synthetic biology where computational RNA sequence design can be used to predictably fine-tune protein production and inserted into construct 5′UTRs. As stronger hairpin structures are introduced, expression levels decrease due to the RNA structure blocking the scanning of the yeast translational machinery during translation initiation. Corroborating this finding, our qPCR experiments showed no difference in transcript levels when mRNAs contained highly-structured or weakly-structured 5′UTRs, and yet these mRNAs expressed protein at greatly different levels. Our method makes control of mRNA translation rates more accessible as a synthetic regulation strategy and importantly requires only a few cloning steps and can be incorporated as a routine part of modular gene construction and optimization alongside the use of promoter libraries that modify mRNA levels (37, 41) and 3′UTR libraries and modules that alter mRNA degradation rates (30, 37).

Accompanying this method, we provide a design script to aid in generating desired hairpin module libraries and predicting their expected expression profiles. This provides users with degenerate-nucleotide encoding sequences that can be used to create the designed hairpin libraries via one-pot cloning methods. The design method avoids the incorporation of premature AUG start codons but does not take into account other possible sequence changes that may change mRNA translation, localization, stability or could affect the upstream promoter by altering nucleosome positioning on the encoding DNA. Non-AUG start codons are also not discounted but are notably present at a significant number of translation start sites in mammalian systems (42, 43). Interestingly, we also uncovered that one class of hairpin structure, the tetraloop motif, reduced gene expression much more than their net MFE contribution would predict (Supplementary Figure S5). This finding requires more investigation, but could be explained if this motif is stabilized by being bound by proteins present during translation initiation. Indeed, tetraloops are known to be conserved folding motifs found in ribosomal RNA, bound by ribosome-associated proteins (44, 45). For now, we recommend not to use tetraloops with our method.

The lack of a full mechanistic understanding of how different 5′UTR structures and sequences affect eukaryotic translation initiation is why we adopted a library approach in this study. While our method cannot guarantee perfect predictability at the level of individual designs, having a library to choose from will almost always yield at least one yeast colony exhibiting the desired expression level. However, for direct design of individual constructs known hairpin sequences from this work can be selected from a pre-characterized list of 5′UTRs shown in Supplementary Tables S5–S8. As others adopt the method, this list will grow and will also provide further data that can be used to improve the sequence-to-expression modeling.

The model developed here, while showing good predictive power, was intentionally simplistic given our limited quantitative understanding of translation initiation processes. But in the future a 5′UTR design tool for yeast (and other eukaryotes) would ideally be based on a model derived from first principles to consider the key biophysical mechanisms at play, just as is done in the bacterial RBS Calculator. The recent success of the Kozak-based yUTR Calculator (18) and a deep-learning based approach to understanding 5′UTR features (19) will no doubt accelerate efforts in this area. But for now, our modular hairpin libraries offer a quick and readily-deployable technology to predictably tune translation, offering up to 100-fold changes in protein levels via insertion of a short sequence away from the boundaries of the promoter and ORF parts.

Our designed hairpins show good predictability in yeast but importantly only ever reduce expression levels (i.e. they cannot be used to increase expression). This is because yeast 5′UTRs rarely contain rate-limiting secondary structures, yet these are seen commonly in other eukaryotes. Indeed, extending our approach to other eukaryotes will require several considerations. A new calibration curve will need to be made for each organism in order to capture differences in behavior of translation initiation machinery. The placement the hairpin module within the 5′UTR may also need to move. In *S. cerevisiae,* we recommend placing the hairpin upstream of the Kozak region for maximum effect, but in higher eukaryotes, the inhibition of translation is thought to be highest when secondary structures are closest to the mRNA 5′ cap (20, 21, 23, 46).

In terms of applications, we anticipate that this approach will be useful broadly but especially in synthetic biology where exploring and tuning gene expression strength is critical. Already a study on gene expression noise in genetic circuits in *S. cerevisiae* has demonstrated the use of 5′UTR hairpins to modify translation rates of a transcript (47). The fact that they can be used to predictably tune expression from promoters without affecting their regulation is especially valuable for those working on gene regulatory circuits and biosensors who require low-leak regulation systems and the ability to *a priori* model expression levels and then achieve these *in vivo.*

We also expect our hairpin library system to be ideal for optimizing the simultaneous expression of multiple genes. Efforts to create heterologous metabolic pathways in yeast are common in synthetic biology and metabolic engineering and efficient ways of optimizing enzyme expression levels in these pathways are needed. Because the fraction of functional library members in a transformed population using our method is high, we expect that multiple libraries can be inserted simultaneously during cloning of a pathway, while at the same time ensuring that functional expression of each gene always occurs.

## Supplementary Material

Supplementary Data
