# Supplementary material for: Design of RNA hairpin modules that predictably tune translation in yeast
Source: Synth Biol (Oxf). 2018 Oct 13;3(1):ysy019. doi: 10.1093/synbio/ysy019 (PMC7445769; doi:10.1093/synbio/ysy019)
Supplement: Supplementary Data [file ysy019_supp.zip › Supplementary.pdf]

Supplementary material: Design of RNA Hairpin Modules that Predictably Tune Translation in Yeast

Tim Weenink, Jelle van der Hilst, Robert M. McKiernan and Tom Ellis

Protocol for the design and construction of 5'UTR hairpin libraries for expression tuning.

The cloning process for the creation of 5'UTR hairpin libraries consists of a small number of simple and rapid cloning steps. The cloning strategy was designed to take 3 days or less for DNA construct preparation or up to 7 days from primer delivery to fully characterised library. The simplicity and efficiency of this method are in large part afforded by use of the Yeast Toolkit (YTK) system and by extension Golden Gate cloning [1]. The few accessory plasmids needed in the cloning process can quickly be generated using the collection of parts included in the YTK.

Figure S1 illustrates the process of plasmid construction in detail. The degenerate hairpin sequence is introduced into the 5'UTR by PCR. The primers used in this study, along with the specific libraries they were used for, are shown in Supplementary table S2. The template is a YTK cassette-level plasmid containing the required promoter and ORF in a transcription unit. Template cassettes used in this study are shown in Supplementary table S3. The PCR product is treated with DpnI to remove the template and subsequently circularised in a Golden Gate reaction with BsaI.

To limit the number of cloning steps and the associated loss in library diversity, the self-ligated PCR product is used directly in the multigene assembly step without passage through *E. coli*, as is customary in the YTK protocol. Equimolar quantities of the product are used in a multigene assembly step, with a pre-assembled yeast integration cassette and a cassette for constitutive mRuby2 expression. The presence of red fluorescent protein is later used as a control for correct plasmid assembly. Consequently, correct clones do not have to be hand-picked during the cloning process, which is unworkable in a library approach with many thousands of individual library members. A list of all libraries created in this study and which cassettes were used in their respective multigene assemblies is provided in Supplementary table S1.

To speed up the process of cloning and to maximise library diversity, the multigene assembly reaction is transformed into the fast growing NEB Turbo competent *E. coli* strain. To allow for parallelization, the transformation is performed with chemically competent cells. The turbo strain can be grown up for miniprep in as little as 5 hours, allowing considerable time savings. To complete the library construction process, the pooled and miniprepped multigene assemblies are digested with NotI in preparation for yeast transformation. The transformation itself is carried out using a high efficiency protocol [2] and a large amount of linearised DNA (3-5 μg) to typically yield thousands of library candidates.

Prior to wet-lab activities, the desired libraries are designed in-silico. Library design starts with the selection of a scaffold hairpin, which represents the strongest structure in the hairpin library space (i.e. the strongest folding library member). The scaffold hairpin must conform to the following design requirements:

- The sequence is entirely or largely palindromic (it is a strong and perfect hairpin).
- The sequence has a minimum free energy of folding that is as low or lower as the lowest required member of the library.
- There is no premature start codon contained in this sequence.
- The loop of the hairpin does not contain an unusually stable sequence known as a tetraloop (see below).
- The two halves of the hairpin are short enough to be contained in the tails of primers used to amplify the selected transcription unit cassette.
- The sequence does not contain BsaI, BsmBI or NotI restriction sites.
- The sequence does not contain other forbidden restriction sites or sequences that interfere with the function of the construct in the chosen application.

In our experiments, the UUCG tetraloop was shown to have a dramatic and unpredictable effect on expression. Inclusion of tetraloop sequences such as GNRA [3, 4], UNCG [3, 5], CUYG [3, 6], UNAC [7] and ANYA [8] is therefore advised against.

Initially the hairpin scaffolds used in this project were based on the ideal Lac operator sequence, which is palindromic. These were subsequently extended and modified to accommodate stronger libraries and shorter cloning primers. However newly designed scaffold can be based on the native 5' UTR - by adding an inverted repeat of this sequence - or on a de-novo structure created through one of many inverse RNA structure prediction solutions available [9, 10, 11].

When a scaffold hairpin has been selected, the degeneracies can be inserted at any base paired position in the hairpin. Substitution of the cytosine in a G-C base pair is preferred, as the opposing guanine is capable of forming the weaker non-canonical G-U pair in addition to the strong canonical pair. This allows variety to be introduced into the hairpin without disrupting its basic structure. In terms of degenerate nucleotide code this is the substitution of the cytosine in a G-C base pair by degenerate nucleotide Y (i.e. C or T). More variety can be created still by also allowing a

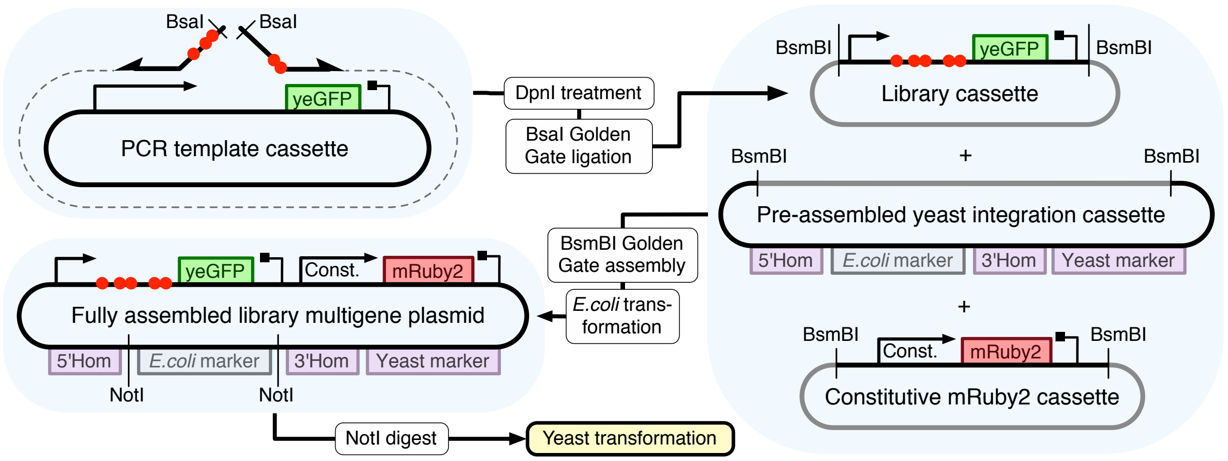

Figure S1: 5'UTR hairpin library construction using the Yeast ToolKit (YTK) system. **1)** Primers encoding the hairpin library of choice (indicated by the red spheres) are used to amplify a template cassette carrying the selected promoter and a green fluorescent reporter gene (yEGFP). A DpnI digestion is performed to eliminate the template and the PCR product is subsequently self-ligated in a BsaI Golden Gate reaction. **2)** The resulting plasmid is purified and directly used in the multigene assembly, bypassing the typical intermediate transformation in order to preserve library diversity. The multigene BsmBI Golden Gate assembly is performed with two additional cassettes: a preassembled yeast integration cassette containing a selectable marker and homology regions for integration, and a constitutively-expressing red fluorescent reporter gene cassette (mRuby2). **3)** NEB turbo competent *E. coli* is transformed with the multigene assembly. The thousands of resulting transformants are pooled and miniprepped. Finally the library of plasmids is digested with NotI before being used in a high efficiency yeast transformation resulting in hundreds to thousands of 5'UTR library candidates.

non-pairing base at this position with degenerate base H (C, T or A). Inclusion of guanine at a locations where it was not originally present is generally avoided because of its large potential for (unintended) interactions. For the same reason, W is the only degenerate nucleotide generally substituted at an A-T pair. Additionally, degeneracies introduced at both sides of the pair are generally avoided, as the probability for a matching basepair decreases drastically as the number of possibilities at each side of the pair increases.

With the scaffold hairpin and degeneracies set, a location in the 5'UTR must be chosen for its insertion. To ensure modularity and conservation of the Kozak sequence, the hairpin is inserted 5-15 bp upstream of the ATG. Further upstream may reduce its effect [12]. To keep the total length of the 5'UTR similar to native 5'UTRs and to ensure the hairpin is not disrupted by unintended basepairing with upstream sequence, the 5' end of the hairpin must be within 10 bp of the transcription start site.

For the next step in the process a fasta file is generated containing all possible sequences that can arise from the selected degeneracies. There are indications that secondary structure involving the start codon has a strong impact on repression. We therefore chose to include the sequence up to that point in the calculations. The cutoff directly after the ATG is still somewhat arbitrary and may be optimised in the future.

The generated list of sequences in multi-FASTA format serves as the input for the RNAfold script used for the folding energy estimation. This is part of the ViennaRNA package 2.0, which is a widely used suite of tools centred around the many facets of RNA structure analysis and prediction [11]. The key non-default parameter used in this script is `-T30`. This modifies the energy parameters used in the script to simulate 30°C, the typical temperature for yeast growth. The full command for script execution is:

```
RNAfold -T30 -d2 --noLP --noPS < Sequence_list_1.fa > Output_MFE_list_1.txt
```

The MFE value for each of the input sequences is then extracted from the output file. This list is used to create a histogram for visualisation of the distribution of folding energies and further to calculate the predicted distribution of expression levels. The logistic fit obtained in **Figure 2** of the main manuscript is used to convert the MFE values to the predicted normalised median fluorescence value for each of the members of the library. Finally, the obtained expression values are plotted as a histogram and compared to the intended expression profile. At this point adjustments can be made to the degeneracies in the hairpin scaffold until the predicted outcome matches the desired outcome.

This pipeline has been implemented in a python script. As input, this script takes the degenerate library sequence and it outputs the associated histograms for the predicted MFE and normalised expression values. It can also output a list of all library member sequences and their associated MFE and expression values. The command for script execution to generate histograms and a complete list of all members for the HL1 library is:

```
python degFoldPlotMod.py -T 30 --prom_strength 210 --output_tsv HL1 AGTATCAACAAAAAgaattgtgagYSYtYatca
gagcgctYaYaattYttTTYgtYGagYAGAAAAACCCCAAATATG
```

The script requires the normalised promoter strength of the promoter that is being used - in this case 210 for the GAL1 promoter - and handles the execution of the RNAfold software without requiring further user interaction. The generated sequences are also analysed for inadvertently introduced premature start codons and restriction sites.

Table S1: Yeast ToolKit multigene level library plasmids with corresponding PCR-generated cassette-level parts. Contrary to normal YTK protocol, one of the cassette-level parts is generated directly with PCR. PCR amplifications were subjected to DpnI treatment and a BsaI-Golden Gate digestion-ligation to circularise the product prior to incorporation into the multigene YTK assembly. Cloned cassette plasmids and template plasmids used in the PCR reactions are detailed in Supplementary table S3.

Library	Description	Cassette 1	Cassette 2	Entry vector	PCR reaction	Forward	Reverse	Template
HL1	pLX-yeGFP -32.2 kcal/mol library	PCR-HL1	T701	pYTK096	PCR-HL1	TW317	TW438	T827
HL2	pLX-yeGFP -28.8 kcal/mol library	PCR-HL2	T701	pYTK096	PCR-HL2	TW317	TW393	T827
HL3	pLX-yeGFP -25.8 kcal/mol library	PCR-HL3	T701	pYTK096	PCR-HL3	TW317	TW392	T827
HL4	pLX-yeGFP -23.4 kcal/mol library	PCR-HL4	T701	pYTK096	PCR-HL4	TW317	TW394	T827
HL5	pLX-yeGFP -20.2kcal/mol library	PCR-HL5	T701	pYTK096	PCR-HL5	TW317	TW323	T827
HL6	pLX-yeGFP -8.0 kcal/mol library	PCR-HL6	T701	pYTK096	PCR-HL6	TW318	TW323	T827
HL1-Red	pLX-mRuby -32.2 kcal/mol library	PCR-HL1-R	T700	pYTK096	PCR-HL1-R	TW411	TW438	C6
HL2-Red	pLX-mRuby -28.8 kcal/mol library	PCR-HL2-R	T700	pYTK096	PCR-HL2-R	TW411	TW393	C6
HL4-Red	pLX-mRuby -23.4 kcal/mol library	PCR-HL4-R	T700	pYTK096	PCR-HL4-R	TW411	TW394	C6
HT1	pLX-yeGFP tetraloop library	PCR-HT1	T701	pYTK096	PCR-HT1	TW317	TW329	T827
HT2	pLX-yeGFP tetraloop library	PCR-HT2	T701	pYTK096	PCR-HT2	TW319	TW330	T827
HT3	pLX-yeGFP tetraloop library	PCR-HT3	T701	pYTK096	PCR-HT3	TW319	TW329	T827
HT4	pLX-yeGFP tetraloop library	PCR-HT4	T701	pYTK096	PCR-HT4	TW318	TW330	T827
HG1	pLX-yeGFP -28.8 kcal/mol library	PCR-HG1	T701	pWS065	PCR-HG1	TW439	TW440	T827
HC1-TDH	pTDH3 -28.8 kcal/mol library	PCR-HC1	T701	pYTK096	PCR-HC1	TW431	TW432	T897
HC1-PGK	pPGK1 -28.8 kcal/mol library	PCR-HC2	T701	pYTK096	PCR-HC2	TW431	TW434	T898
HC1-TEF	pTEF2 -28.8 kcal/mol library	PCR-HC3	T701	pYTK096	PCR-HC3	TW431	TW433	C7
HC1-YRA	pYRA1s -28.8 kcal/mol library	PCR-HC4	T701	pYTK096	PCR-HC4	TW431	TW435	T899
HC1-POP	pPOP6 -28.8 kcal/mol library	PCR-HC5	T701	pYTK096	PCR-HC5	TW431	TW437	T901

Table S2: Primers used in this study. Capital letters indicate annealing sequence and locations of nucleotide degeneracies. W = A or T; H = A, C or T; Y = C or T; R = A or G; D = A, G or T; V = A, C or G; S = G or C; K = G or T

Name	Sequence	Direction	Purpose
TW318	ttttggtctcaagcgctWaHaattAttWWYgtYRagDAGAAAAACCCCAAATATGGTTTC	Fw	HL6
TW317	ttttggtctcaagcgctYaYaattYttTTYgtYGagYAGAAAAACCCCAAATATGGTTTC	Fw	HL5, HL4, HL3, HL2, HL1
TW411	ttttggtctcaagcgctYaYaattYttTTYgtYGagYAGAAAAACCCCAAATATGGT	Fw	HL1-R, HL2-R, HL4-R
TW323	ttttggtctcacgctcacaattcTTTTTGTTGATACTTTTATTACATTTG	Rev	HL6, HL5
TW394	ttttggtctcacgctctgatTaWVTctcacaattcTTTTTGTTGATACTTTTATTACATT	Rev	HL4, HL4-R
TW392	ttttggtctcacgctctgatWaWSWctcacaattcTTTTTGTTGATACTTTTATTACATT	Rev	HL3
TW393	ttttggtctcacgctctgatKaKSKctcacaattcTTTTTGTTGATACTTTTATTACATT	Rev	HL2, HL2-R
TW438	ttttggtctcacgctctgatRaRSRctcacaattcTTTTTGTTGATACTTTTATTACATT	Rev	HL1, HL1-R
TW317	ttttggtctcaagcgctYaYaattYttTTYgtYGagYAGAAAAACCCCAAATATGGTTTC	Fw	HT1
TW318	ttttggtctcaagcgctWaHaattAttWWYgtYRagDAGAAAAACCCCAAATATGGTTTC	Fw	HT4
TW319	ttttggtctcaagcgctYaYaattHttWWYgtYRagSAGAAAAACCCCAAATATGGTTTC	Fw	HT2, HT3
TW329	ttttggtctcacgctccgaaRaRSRctcacaattcTTTTTGTTGATACTTTTATTACATT	Rev	HT1, HT3
TW330	ttttggtctcacgctccgaaGaGCGctcacaattcTTTTTGTTGATACTTTTATTACATT	Rev	HT2, HT4
TW439	ttttggtctcaagcgcagtgttaagttTTYgtYGagYAGAAAAACCCCAAATATGGT	Fw	HG1 Gal repression lib.
TW440	ttttggtctcaCGCTctgatWaWSWcaRtRttaaRTTTTTGTTGATACTTTTATTACATT	Rev	HG1 Gal repression lib.
TW431	ttttggtctcaatcagagHgYtYaYaattYttaaRgaAGATCTATGGTTTCTAAAGGTGA	Fw	All constitutive libraries
TW432	ttttggtctcatgatRaRcRctcacaattcttaaagaTTTGTTTGTTTATGTGTGTTTAT	Rev	HC1-TDH3
TW434	ttttggtctcatgatRaRcRctcacaattcttaaagaTGTTTTATATTTGTTGTAAAAAG	Rev	HC1-PGK1
TW433	ttttggtctcatgatRaRcRctcacaattcttaaagaGTTTAGTTAATTATAGTTCGTTG	Rev	HC1-TEF2
TW435	ttttggtctcatgatRaRcRctcacaattcttaaagaTTAGCAGATGTAGGTATTTTCTT	Rev	HC1-YRA1s
TW437	ttttggtctcatgatRaRcRctcacaattcttaaagaTTTGATTTGCTTTTATCTTTTTT	Rev	HC1-POP6
TW188	GAAGTACGGATTAGAAGCCG	Fw	Col.PCR yEGFP, mRuby2
TW149	CAGCTCTGGTCTTGTAGTTAC	Rev	Col.PCR yEGFP
TW195	CATTGAACACCATAACCGAAAG	Rev	Sequencing yEGFP
TW325	CCATCAGCTGGGTACATCAT	Rev	Col.PCR mRuby2
TW457	CATCTTCGTATCTTGTAAC	Rev	Sequencing mRuby2
TW164	GTGTCGGTGTCATCTTGTGT	Fw	qPCR TPI1
TW165	ACGACGTTAGTCCAGTCCTT	Rev	qPCR and RT TPI1
TW167	CATTATCGTTGGGCTGGTCT	Rev	RT DOA1
TW168	GGTGATGGTCCAGTCTTGTT	Fw	qPCR yeGFP
TW169	ATGGGTAATACCAGCAGCAG	Rev	qPCR and RT yeGFP
TW178	GTCAGCGACAACCCATATAC	Fw	qPCR DOA1
TW179	CTGGTCTAGCGATATGCCATT	Rev	qPCR DOA1

Table S3: Yeast ToolKit cassette-level plasmids used in the construction process, as template for PCR or directly in a multigene-assembly step. Part-level plasmids used in the assembly of these cassettes are listed. Sequences for part-level plasmids not included in the YTK are given in Supplementary table S4.

Name	Description	Parts used in assembly						
T827	pGAL1-yEGFP cassette	pYTK002	T655	T675	pTMP065	pYTK068	pYTK095	
C6	pGAL1-mRuby2 cassette	pYTK002	T655	pYKT034	pTMP065	pYTK068	pYTK095	
T897	pTDH3-yEGFP cassette	pYTK002	pYTK009	T675	pTMP065	pYTK068	pYTK095	
C7	pTEF2-yEGFP cassette	pYTK002	pYTK014	T675	pTMP065	pYTK068	pYTK095	
T898	pPGK1-yEGFP cassette	pYTK002	pTMP030	T675	pTMP065	pYTK068	pYTK095	
T899	pYRA1s-yEGFP cassette	pYTK002	pYTK018	T675	pTMP065	pYTK068	pYTK095	
T901	pPOP6-yEGFP cassette	pYTK002	pYTK024	T675	pTMP065	pYTK068	pYTK095	
T700	pTEF-yEGFP cassette	pYTK004	pYTK013	T675	pYTK054	pYTK072	pYTK095	
T701	pTEF-mRuby2 cassette	pYTK004	pYTK013	pYTK034	pYTK054	pYTK072	pYTK095	
pWS065	pre-assembled HIS integration cassette	pYTK008	pYTK047	pYTK094	pYTK073	pYTK076	pYTK088	pYTK090

Table S4: Custom part-level plasmids for the Yeast ToolKit used for the creation of 5'UTR hairpin libraries. The standard backbone sequence for YTK part-level plasmids is not shown.

Name	Description	Part type	Part sequence including BsmBI sites
T655	pLX Gal1 derived LacI repressible promoter	2	GGTCTCAAACGGAAGTACGGATTAGAAGCCGCCGAGCGGGTGACAGCCCTCCGAAGGAAGACTCTCCTCCGTGCGTCCTCGTCTTCACCGGTCGCGTT<br>CCTGAAACGCAGATGTGCCTCGCGCCGCACTGCTCCGAACAATAAAGATTCTACAATACTAGCTTTTATGGTTATGAAGAGGAAAAATTGGCAGTAAC<br>CTGGCCCCACAAACCTTCAAATGAACGAATCAAATTAACAACCCTAGGATGATAATGCGATTACTTTTTTAGCCTTATTTCTGGGGTACTGCAGCAGC<br>GAAGCGATGATTTTTGATCTATTAACAGATATATAAATGCAAAAACTGTTGTTGTGTGGAATTGTGAGCGGATAACAATTTCACACAATATTACTTCT<br>TATTCAAATGTAATAAAAGTATCAACAAAAAATTGTTAATATACCTCTATACTTTAACGTCAAGGAGAAAAAACCCCAAATATGTGAGACC
T675	yEGFP	3	GGTCTCATATGGTTTCTAAAGGTGAAGAATTATTCACTGGTGTTGTCCCAATTTTGGTTGAATTAGATGGTGATGTTAATGGTCACAAATTTTCTGTC<br>TCCGGTGAAGGTGAAGGTGATGCTACTTACGGTAAATTGACCTTAAAATTTATTTGTACTACTGGTAAATTGCCAGTTCCATGGCCAACCTTAGTCAC<br>TACTTTCGGTTATGGTGTTCAATGTTTTGCTAGATACCCAGATCATATGAAACAACATGACTTTTTCAAGTCTGCCATGCCAGAAGGTTATGTTCAAG<br>AAAGAACTATTTTTTTCAAAGATGACGGTAACTACAAGACCAGAGCTGAAGTCAAGTTTGAAGGTGATACCTTAGTTAATAGAATCGAATTAAAAGGT<br>ATTGATTTTAAAGAAGATGGTAACATTTTAGGTCACAAATTGGAATACAACTATAACTCTCACAATGTTTACATCATGGCTGACAAACAAAAGAATGG<br>TATCAAAGTTAACTTCAAAATTAGACACAACATTGAAGATGGTTCTGTTCAATTAGCTGACCATTATCAACAAAATACTCCAATTGGTGATGGTCCAG<br>TCTTGTTACCAGACAACCATTACTTATCCACTCAATCTGCCTTATCCAAAGATCCAAACGAAAAGAGAGATCACATGGTCTTGTTAGAATTTGTTACT<br>GCTGCTGGTATTACCCATGGTATGGATGAATTGTACAAAGGATCCTGAGACC
pTMP030	pYRA1s promoter	2	GGTCTCAAACGAAACTTGTGGGCGCAATTATAAAACACTGCTACCAATTGTTCGTTTTCTGTTCATTAACACATAAAAAACCCTTATGTAACTATATT<br>TACAAAGTAAATACGTATATTAAAGCTATTTTACCACTACCACAGAGTTCTTTGTCCAGTTGCTAGTATTTTTTTTTTCGCGACGAGGCAGGGGCGGG<br>TAGACGTGTTGTTTTTCCACGGCTTTCGGCTCACCACTTGAAGAACTATAAAAGGCCGCCAAATTTATCCTTTTTCACTTCTTCCGTTCGCTTTTTTC<br>TGTCATTCCTATCGTGTGTTTAGTAGTAGGTTTTTTTGTTAGAAGAAGTTTTATCCGAAAACTATCGAtGACAAATAGATAAAAAAATCTCCCTCGTT<br>CTATTTGAAACTTTAAGAAATCCATATTAAGAAAATACCTACATCTGCTAAAGATCTATGTGAGACC
pTMP065	tPDC1 terminator	4	GGTCTCAATCCTAACTCGAGGCGATTTAATCTCTAATTATTAGTTAAAGTTTTATAAGCATTTTTATGTAACGAAAAATAAATTGGTTCATATTATTA<br>CTGCACTGTCACTTACCATGGAAAGACCAGACAAGAAGTTGCCGACAGTCTGTTGAATTGGCCTGGTTAGGCTTAAGTCTGGGTCCGCTTCTTTACAA<br>ATTTGGAGAATTTCTCTTAAACGATATGTATATTCTTTTCGTTGGAAAAGCTGTGAGACC

Table S5: List of all library isolates displayed in figure 2A. For each isolate, the following properties are listed: parental library, normalised median fluorescence, standard deviation of the fluorescence over three biological replicates, predicted minimum free energy of folding of the 5'UTR and its DNA sequence.

Isolate	normalised fluorescence	standard deviation	MFE (kcal/mol)	Sequence
Original promoter	198	4.60	-7.81	AGTATCAACAAAAAATTGTTAATATACCTCTATACTTTAACGTCAAGGAGAAAAACCCCaaatatg
HL6a	239	3.95	-7.22	AGTATCAACAAAAAgaattgtgagcgctTaCaattAttAATgtCAagAAGAAAAACCCCAAATATG
HL2a	150	6.52	-22.74	AGTATCAACAAAAAGAATTGTGAGACATAATCAGAGCGCTTATAATTTTTTTGTTGAGTAGAAAAACCCCAAATATG
HL2b	125	7.06	-20.08	AGTATCAACAAAAATTTAACATTGACATAATCAGAGCGCAGTGTTAAGTTTTCGTTGAGTAGAAAAACCCCAAATATG
HL2c	119	4.82	-24.06	AGTATCAACAAAAAGAATTGTGAGAGATCATCAGAGCGCTCATAATTTTTTCGTTGAGTAGAAAAACCCCAAATATG
HL2d	124	7.55	-24.64	AGTATCAACAAAAAGAATTGTGAGACCTCATCAGAGCGCTTATAATTTTTTCGTTGAGTAGAAAAACCCCAAATATG
HL2e	39.2	2.46	-26.95	AGTATCAACAAAAAGAATTGTGAGACATAATCAGAGCGCTCACAATTTTTTTGTTGAGTAGAAAAACCCCAAATATG
HL2f	3.58	0.23	-38.55	AGTATCAACAAAAAGAATTGTGAGCGCTAATCAGAGCGCTTACAATTTTTTTGTTGAGCAGAAAAACCCCAAATATG
HL2g	9.62	0.79	-34.2	AGTATCAACAAAAAGAATTGTGAGCGCTAATCAGAGCGCTTACAATTTTTTCGTTGAGCAGAAAAACCCCAAATATG
HL2h	124	4.04	-22.18	AGTATCAACAAAAAGAATTGTGAGACATCATCAGAGCGCTTACAATTCTTTTCGTCGAGTAGAAAAACCCCAAATATG
HL2i	2.62	0.13	-39.07	AGTATCAACAAAAAGAATTGTGAGCGCTCATCAGAGCGCTTATAATTTTTTTGTTGAGTAGAAAAACCCCAAATATG
HL2j	25.4	0.84	-30.88	AGTATCAACAAAAAGAATTGTGAGACATCATCAGAGCGCTTACAATTCTTTTTGTTGAGTAGAAAAACCCCAAATATG
HL2k	143	7.77	-24.54	AGTATCAACAAAAAGAATTGTGAGAGATCATCAGAGCGCTTATAATTCTTTTCGTTGAGTAGAAAAACCCCAAATATG
HL2l	52.0	2.92	-25.49	AGTATCAACAAAAAGAATTGTGAGCCCTAATCAGAGCGCTCATAATTCTTTTCGTCGAGTAGAAAAACCCCAAATATG
HL2m	53.9	2.74	-24.12	AGTATCAACAAAAAGAATTGTGAGACATAATCAGAGCGCTCATAATTCTTTTTGTCGAGCAGAAAAACCCCAAATATG
HL2n	109	7.54	-27.62	AGTATCAACAAAAAGAATTGTGAGCCCTAATCAGAGCGCTTATAATTCTTTTCGTTGAGTAGAAAAACCCCAAATATG
HL2o	89.8	8.67	-25.53	AGTATCAACAAAAAGAATTGTGAGACCTAATCAGAGCGCTCACAATTTTTTTGTCGAGTAGAAAAACCCCAAATATG
HL2p	178	11.6	-13.97	AGTATCAACAAAAAGAATTGTGAGCCATAATCAGAGCG–TCATAATTTTTTTT–CGAGTAGAAAAACCCCAAATATG
HL4a	185	10.1	-18.59	AGTATCAACAAAAAGAATTGTGAGAGTTAATCAGAGCGCTTATAATTTTTTCGTCGAGTAGAAAAACCCCAAATATG
HL1a	7.32	0.04	-35.64	AGTATCAACAAAAAGAATTGTGAGTGTTCATCAGAGCGCTTACAATTCTTTTTGTCGAGCAGAAAAACCCCAAATATG
HL1b	2.71	0.40	-36.37	AGTATCAACAAAAAGAATTGTGAGCGTTCATCAGAGCGCTCACAATTTTTTTGTCGAGTAGAAAAACCCCAAATATG
HL1c	4.64	0.16	-40.55	AGTATCAACAAAAAGAATTGTGAGTGCTTATCAGAGCGCTCATAATTCTTTTTGTTGAGCAGAAAAACCCCAAATATG
HL1d	22.4	0.65	-33.27	AGTATCAACAAAAAGAATTGTGAGTGCTTATCAGAGCGCTTACAATTTTTTTGTCGAGCAGAAAAACCCCAAATATG
HL1e	22.6	0.55	-31.44	AGTATCAACAAAAAGAATTGTGAGTCCTCATCAGAGCGCTCATAATTCTTTTTGTCGAGTAGAAAAACCCCAAATATG
HL1f	9.81	0.28	-32.73	AGTATCAACAAAAAGAATTGTGAGCGTTCATCAGAGCGCTCATAATTCTTTTCGTCGAGTAGAAAAACCCCAAATATG
HL1g	33.8	1.60	-30.46	AGTATCAACAAAAAGAATTGTGAGCCCTCATCAGAGCGCTCATAATTTTTTTGTCGAGTAGAAAAACCCCAAATATG
HL1h	1.92	0.24	-42.54	AGTATCAACAAAAAGAATTGTGAGTGCTTATCAGAGCGCTCACAATTCTTTTTGTTGAGCAGAAAAACCCCAAATATG
HL1i	8.86	0.33	-34.57	AGTATCAACAAAAAGAATTGTGAGCCCTTATCAGAGCGCTCACAATTTTTTTGTTGAGTAGAAAAACCCCAAATATG
HL1j	11.9	1.03	-30.46	AGTATCAACAAAAAGAATTGTGAGC–CTCATCAGAGCGCTCATAATTTTTTTGTCGAGTAGAAAAACCCCAAATATG
HL1k	1.58	0.17	-40.21	AGTATCAACAAAAAGAATTGTGAGCGCTTATCAGAGCGCTCACAATTCTTTTTGTCGAGCAGAAAAACCCCAAATATG
HL1l	1.46	0.26	-41.86	AGTATCAACAAAAAGAATTGTGAGCGCTTATCAGAGCGCTCACAATTTTTTTGTTGAGCAGAAAAACCCCAAATATG
HL1m	7.62	0.79	-35.19	AGTATCAACAAAAAGAATTGTGAGCGTTCATCAGAGCGCTCATAATTTTTTTGTCGAGCAGAAAAACCCCAAATATG

Table S6: List of all library isolates displayed in figure 3A. For each isolate, the following properties are listed: parental library, normalised median fluorescence, standard deviation of the fluorescence over three biological replicates, predicted minimum free energy of folding of the 5'UTR and its DNA sequence.

Isolate	normalised fluorescence	standard deviation	MFE (kcal/mol)	Sequence
Original promoter	178.0	15.7	-7.81	AATAAAAGTATCAACAAAAAATTGTTAATATACCTCTATACTTTAACGTCAAGGAGAAAAACCCCaaatatg
HL3ga	125.8	18.4	-20.75	AATAAAAGTATCAACAAAAAGAATTGTGAGTCTTTATCAGAGCGCTCATAATTTTTTCGTCGAGTAGAAAAACCCCAAATATG
HL3gb	116.2	15.9	-20.95	AATAAAAGTATCAACAAAAAGAATTGTGAGACTTTATCAGAGCGCTTATAATTTTTTTGTCGAGCAGAAAAACCCCAAATATG
HL3gc	98.9	18.5	-18.06	AATAAAAGTATCAACAAAAAGAATTGTGAGACATTATCAGAGCGCTTACAATTTTTTCGTCGAGCAGAAAAACCCCAAATATG
HL3gd	90.2	21.0	-17.55	AATAAAAGTATCAACAAAAAGAATTGTGAGACATAATCAGAGCGCTTATAATTCTTTTCGTCGAGCAGAAAAACCCCAAATATG
HL3ge	84.5	19.1	-23.75	AATAAAAGTATCAACAAAAAGAATTGTGAGAGTTAATCAGAGCGCTTATAATTTTTTTGTCGAGCAGAAAAACCCCAAATATG
HL3gf	60.9	9.2	-28.12	AATAAAAGTATCAACAAAAAGAATTGTGAGTCTTAATCAGAGCGCTTATAATTTTTTTGTTGAGCAGAAAAACCCCAAATATG
HL3gg	45.6	5.1	-26.25	AATAAAAGTATCAACAAAAAGAATTGTGAGACATAATCAGAGCGCTTATAATTCTTTTTGTTGAGCAGAAAAACCCCAAATATG
HL3gh	34.4	5.8	-25.76	AATAAAAGTATCAACAAAAAGAATTGTGAGTCATAATCAGAGCGCTCATAATTCTTTTCGTTGAGCAGAAAAACCCCAAATATG
HL3gi	27.3	4.1	-25.44	AATAAAAGTATCAACAAAAAGAATTGTGAGTCTTTATCAGAGCGCTCACAATTCTTTTCGTCGAGTAGAAAAACCCCAAATATG
HL3gj	20.6	4.6	-29.05	AATAAAAGTATCAACAAAAAGAATTGTGAGAGTTTATCAGAGCGCTCACAATTTTTTCGTTGAGCAGAAAAACCCCAAATATG
HL3gk	14.5	2.1	-33.97	AATAAAAGTATCAACAAAAAGAATTGTGAGTGTTAATCAGAGCGCTTACAATTTTTTTGTTGAGCAGAAAAACCCCAAATATG
HL3gl	10.5	1.7	-32.25	AATAAAAGTATCAACAAAAAGAATTGTGAGTCTTTATCAGAGCGCTCACAATTTTTTTGTTGAGCAGAAAAACCCCAAATATG
HL3gm	8.9	0.86	-29.99	AATAAAAGTATCAACAAAAAGAATTGTGAGACTTTATCAGAGCGCTTACAATTCTTTTTGTTGAGCAGAAAAACCCCAAATATG
HL3gn	8.0	1.2	-31.68	AATAAAAGTATCAACAAAAAGAATTGTGAGAGATTATCAGAGCGCTCACAATTCTTTTTGTTGAGCAGAAAAACCCCAAATATG
HL3go	4.8	1.9	-36.47	AATAAAAGTATCAACAAAAAGAATTGTGAGTGTTTATCAGAGCGCTCACAATTTTTTTGTTGAGTAGAAAAACCCCAAATATG
HL3gp	0.81	0.19	-31.44	AATAAAAGTATCAACAAAAAGAATTGTGAGTCTTTATCAGAGCGCTCACAATTTTTTTGTTGAGTAGAAAAACCCCAAATATG

Table S7: List of all library isolates displayed in figure 3B. For each isolate, the following properties are listed: parental library, normalised median fluorescence, standard deviation of the fluorescence over three biological replicates, predicted minimum free energy of folding of the 5'UTR and its DNA sequence.

Isolate	normalised fluorescence	standard deviation	MFE (kcal/mol)	Sequence
Original promoter	41.99	1.22	-7.81	AATAAAAGTATCAACAAAAAATTGTTAATATACCTCTATACTTTAACGTCAAGGAGAAAAACCCCaaatatg
HL1ra	32.22	2.09	-13.89	AATAAAAGTATCAACAAAAAGAATTGTGA-TCTTCATCAGAGCGCTTATAATTTTTTTCGTCGAGCAGAAAAACCCCAAATATG
HL1rb	28.80	2.34	-19.74	AATAAAAGTATCAACAAAAAGAATTGTGAGTCTTCATCAGAGCGCTTATAATTTTTTTCGTCGAGTAGAAAAACCCCAAATATG
HL1rc	28.11	4.56	-26.82	AATAAAAGTATCAACAAAAAGAATTGTGAGCCCTTATCAGAGCGCTTATAATTTTTTTCGTTGAGCAGAAAAACCCCAAATATG
HL1rd	14.18	0.76	-27.33	AATAAAAGTATCAACAAAAAGAATTGTGAGTCCTCATCAGAGCGCTTATAATTTTTTTTGTCGAGCAGAAAAACCCCAAATATG
HL1re	6.45	0.29	-32.02	AATAAAAGTATCAACAAAAAGAATTGTGAGCCTTTATCAGAGCGCTTACAATTTTTTTTGTTGAGTAGAAAAACCCCAAATATG
HL1rf	5.22	0.68	-30.5	AATAAAAGTATCAACAAAAAGAATTGTGAGCGTTTATCAGAGCGCTCATAATTCTTTTCGTCGAGTAGAAAAACCCCAAATATG
HL1rg	3.50	0.19	-32.02	AATAAAAGTATCAACAAAAAGAATTGTGAGTCCTCATCAGAGCGCTTACAATTCTTTTCGTTGAGCAGAAAAACCCCAAATATG
HL1rh	2.35	0.18	-35.39	AATAAAAGTATCAACAAAAAGAATTGTGAGCCCTCATCAGAGCGCTTACAATTTTTTTTGTTGAGCAGAAAAACCCCAAATATG
HL1ri	1.59	0.041	-33.26	AATAAAAGTATCAACAAAAAGAATTGTGAGCCCTCATCAGAGCGCTCACAATTTTTTTCGTTGAGCAGAAAAACCCCAAATATG
HL1rj	1.35	0.018	-33.26	AATAAAAGTATCAACAAAAAGAATTGTGAGCCCTCATCAGAGCGCTCACAATTTTTTTCGTTGAGCAGAAAAACCCCAAATATG
HL1rk	1.13	0.011	-33.85	AATAAAAGTATCAACAAAAAGAATTGTGAGTGCTCATCAGAGCGCTTACAATTCTTTTCGTCGAGCAGAAAAACCCCAAATATG
HL1rl	1.07	0.021	-37.72	AATAAAAGTATCAACAAAAAGAATTGTGAGTGCTCATCAGAGCGCTCACAATTTTTTTCGTTGAGCAGAAAAACCCCAAATATG
HL1rm	1.07	0.013	-35.86	AATAAAAGTATCAACAAAAAGAATTGTGAGCGCTTATCAGAGCGCTCACAATTCTTTTCGTCGAGCAGAAAAACCCCAAATATG

Table S8: List of all library isolates displayed in figure 3C. For each isolate, the following properties are listed: parental library, normalised median fluorescence, standard deviation of the fluorescence over three biological replicates, predicted minimum free energy of folding of the 5'UTR and its DNA sequence.

Isolate	normalised fluorescence	standard deviation	MFE (kcal/mol)	Sequence
original promoter	59.2	5.76	-5.78	tcaaggaagtaattatctacttttttacaacaaatataaaacaagatctatg
PGK1HC1a	35.6	5.53	-24.9	TCAAGGAAGTAATTATCTACTTTTTACAACAAATATAAAACATCTTTAAGAATTGTGAGTGCTTATCAGAGAGTTTATAATTTTTAAGGAAGATCTATG
PGK1HC1b	30.9	3.23	-23.54	TCAAGGAAGTAATTATCTACTTTTTACAACAAATATAAAACATCTTTAAGAATTGTGAGTGTTTATCAGAGAGTTCATAATTTTTAAGGAAGATCTATG
PGK1HC1c	26.8	4.43	-27.12	TCAAGGAAGTAATTATCTACTTTTTACAACAAATATAAAACATCTTTAAGAATTGTGAGCGTTTATCAGAGTGTTTATAATTTTTAAGGAAGATCTATG
PGK1HC1d	15.8	1.29	-29.44	TCAAGGAAGTAATTATCTACTTTTTACAACAAATATAAAACATCTTTAAGAATTGTGAGCGTTTATCAGAGAGTTCATAATTTTTAAAGAAGATCTATG
PGK1HC1e	11.4	1.07	-29.11	TCAAGGAAGTAATTATCTACTTTTTACAACAAATATAAAACATCTTTAAGAATTGTGAGTGTTTATCAGAGCGTTTACAATTTTTAAGGAAGATCTATG
PGK1HC1f	8.4	0.77	-29.68	TCAAGGAAGTAATTATCTACTTTTTACAACAAATATAAAACATCTTTAAGAATTGTGAGTGCTTATCAGAGCGTTTATAATTTTTAAGGAAGATCTATG
PGK1HC1g	6.1	0.67	-34.97	TCAAGGAAGTAATTATCTACTTTTTACAACAAATATAAAACATCTTTAAGAATTGTGAGCGCTTATCAGAGTGTTCATAATTTTTAAAGAAGATCTATG
PGK1HC1h	4.3	0.43	-35.68	TCAAGGAAGTAATTATCTACTTTTTACAACAAATATAAAACATCTTTAAGAATTGTGAGCGCTCATCAGAGAGTTCATAATTCTTAAGGAAGATCTATG
PGK1HC1i	2.9	0.34	-34.64	TCAAGGAAGTAATTATCTACTTTTTACAACAAATATAAAACATCTTTAAGAATTGTGAGCGCTCATCAGAGTGTTTATAATTTTTAAGGAAGATCTATG
PGK1HC1j	2.4	0.24	-36.76	TCAAGGAAGTAATTATCTACTTTTTACAACAAATATAAAACATCTTTAAGAATTGTGAGCGCTTATCAGAGCGTTTACAATTTTTAAAGAAGATCTATG
PGK1HC1k	2.0	0.26	-36.93	TCAAGGAAGTAATTATCTACTTTTTACAACAAATATAAAACATCTTTAAGAATTGTGAGTGCTTATCAGAGCGTTCACAATTCTTAAAGAAGATCTATG
PGK1HC1l	1.7	0.23	-38.35	TCAAGGAAGTAATTATCTACTTTTTACAACAAATATAAAACATCTTTAAGAATTGTGAGCGCTCATCAGAGAGCTTACAATTCTTAAAGAAGATCTATG
PGK1HC1m	1.3	0.21	-38.85	TCAAGGAAGTAATTATCTACTTTTTACAACAAATATAAAACATCTTTAAGAATTGTGAGCGCTCATCAGAGTGTTCACAATTTTTAAGGAAGATCTATG
PGK1HC1n	1.1	0.20	-41.21	TCAAGGAAGTAATTATCTACTTTTTACAACAAATATAAAACATCTTTAAGAATTGTGAGCGTTCATCAGAGCGCTCACAATTTTTAAAGAAGATCTATG
PGK1HC1o	1.0	0.19	-41.21	TCAAGGAAGTAATTATCTACTTTTTACAACAAATATAAAACATCTTTAAGAATTGTGAGCGCTCATCAGAGCGTTCACAATTTTTAAAGAAGATCTATG
PGK1HC1p	0.96	0.18	-41.89	TCAAGGAAGTAATTATCTACTTTTTACAACAAATATAAAACATCTTTAAGAATTGTGAGCGCTCATCAGAGTGTTCACAATTCTTAAAGAAGATCTATG
PGK1HC1q	0.93	0.18	-41.35	TCAAGGAAGTAATTATCTACTTTTTACAACAAATATAAAACATCTTTAAGAATTGTGAGCGCTCATCAGAGCGTTTACAATTCTTAAGGAAGATCTATG

Table S9: Sequences of the various hairpin libraries tested in this study. Hairpin stems are annotated in red, tetraloops in blue and the start codon in green. MFE in kcal/mol indicates the average minimum free energy of folding of each of the hairpin libraries.

Library name	Library sequence	MFE in kcal/mol
HL1	AGTATCAACAAAAAGaattgtgagYSYtYatcagagcgctYaYaattYttTTYgtYGagYAGAAAAACCCCAAATATG	-32.2
HL2	AGTATCAACAAAAAGaattgtgagMSMtMatcagagcgctYaYaattYttTTYgtYGagYAGAAAAACCCCAAATATG	-28.8
HL3	AGTATCAACAAAAAGaattgtgagWSWtWatcagagcgctYaYaattYttTTYgtYGagYAGAAAAACCCCAAATATG	-25.8
HL4	AGTATCAACAAAAAGaattgtgagABWtAatcagagcgctYaYaattYttTTYgtYGagYAGAAAAACCCCAAATATG	-23.4
HL5	AGTATCAACAAAAAGaattgtgagcgctYaYaattYttTTYgtYGagYAGAAAAACCCCAAATATG	-20.2
HL6	AGTATCAACAAAAAGaattgtgagcgctWaHaattAttWWYgtYRagDAGAAAAACCCCAAATATG	-8.0
HT1	AGTATCAACAAAAAGaattgtgagYSYtYTTCGgagcgctYaYaattYttTTYgtYGagYAGAAAAACCCCAAATATG	-32.5
HT2	AGTATCAACAAAAAGaattgtgagCGCtCTTCGgagcgctYaYaattHttWWYgtYRagSAGAAAAACCCCAAATATG	-33.2
HT3	AGTATCAACAAAAAGaattgtgagYSYtYTTCGgagcgctYaYaattHttWWYgtYRagSAGAAAAACCCCAAATATG	-26.5
HT4	AGTATCAACAAAAAGaattgtgagCGCtCTTCGgagcgctWaHaattAttWWYgtYRagDAGAAAAACCCCAAATATG	-24.8
HG1	AGTATCAACAAAAAYttaaYaYtgWSWtWatcagAGCGcagtgttaagttTTYgtYGagYAGAAAAACCCCAAATATG	-28.8
HC1	tctttaagaattgtgagYgYtYatcagagHgYtYaYaattYttaaRgaAGATCTATG	-28.9

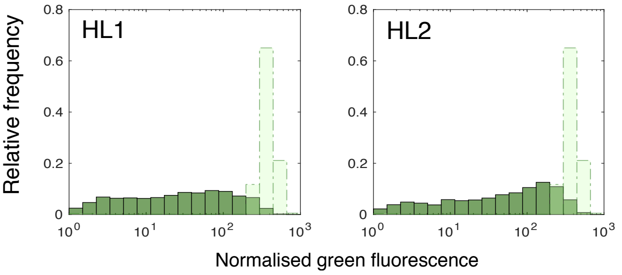

Figure S2: For reference, figure panel 1D from the main paper is reproduced here as plotted on single y-axes. The histogram of normalised fluorescence from library HL1 (left) and HL2 (right) is shown in dark green. The histogram of normalised fluorescence from the original GAL1 promoter is shown in light green with dashed outline.

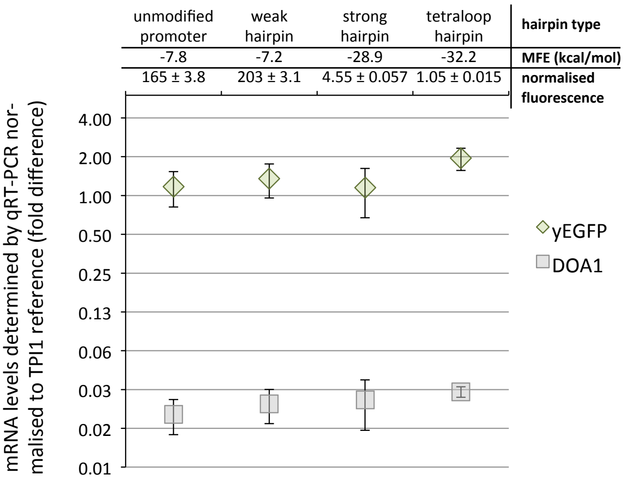

Figure S3: Low impact of 5'UTR structure on transcript levels per cell. Two-step qPCR was used to determine transcript levels in strains with hairpins of various strengths in the 5'UTR of yEGFP expressed from a pLX GAL1-derived promoter. Associated folding energies (Minimum Free Energy, MFE in kcal/mol) and normalised expression strength of each construct is shown at the top of the graph. Note that mRNA levels are not affected by changes in expression strength between the constructs. Expression strengths are normalised against cellular autofluorescence of the parental strain. Transcript levels are normalised against the TPI1 mRNA, using the dd-Ct method [13]. TPI1 is a strongly expressed reference gene (˜200 mRNAs per cell), while DOA1 is a weakly expressed reference gene (2.6 mRNAs per cell on average). Error bars indicate standard deviation of technical triplicates.

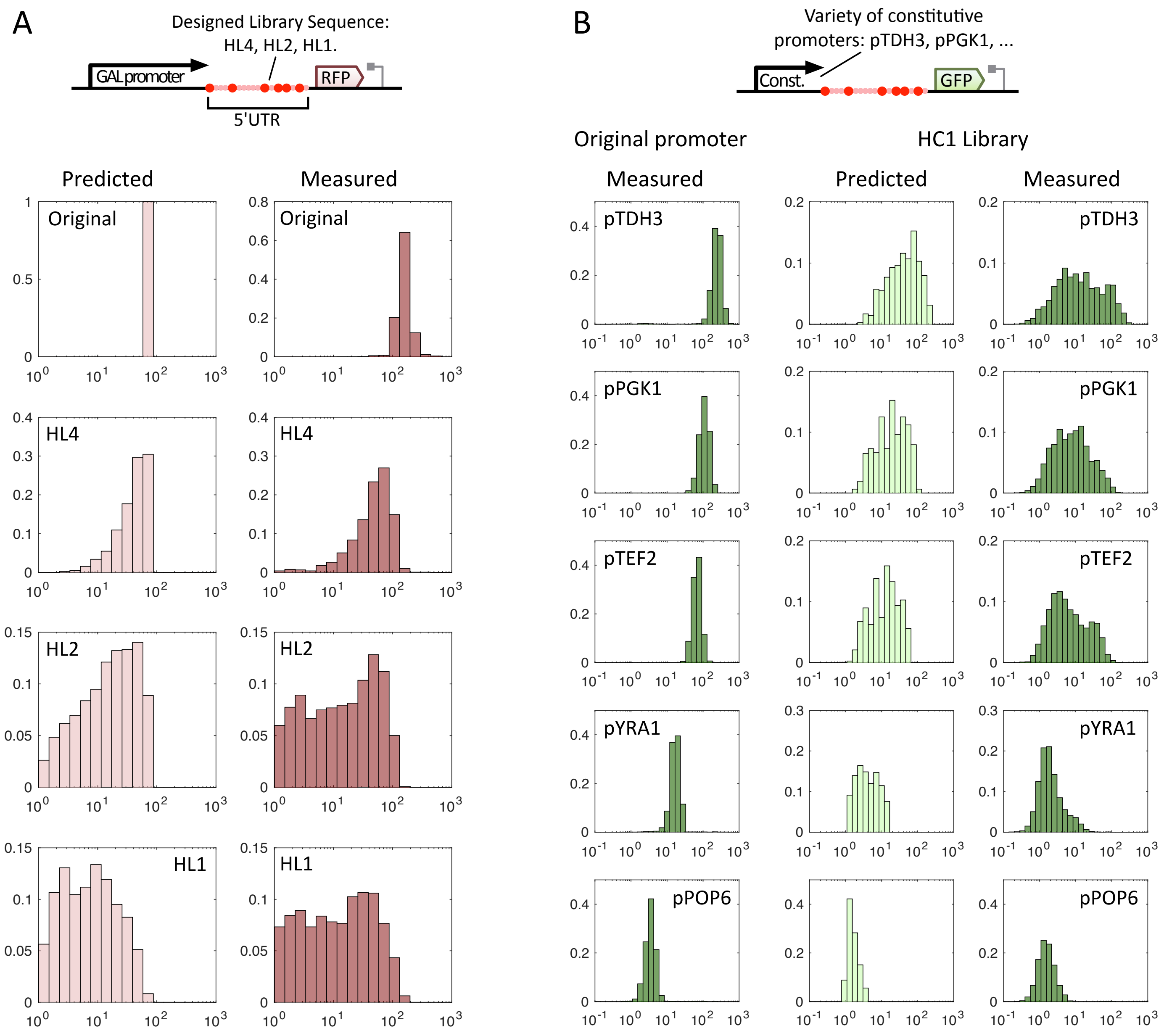

Figure S4: Robustness of predictions with respect to upstream and downstream sequence. Distributions are shown as histograms with normalised fluorescence logarithmically on the x-axis and relative frequency linearly on the y-axis, both unit-less quantities. MFE stands for minimum free energy of folding, a measure of the strength of the hairpin structure in the 5'UTR. (**A**) Robustness of the library predictions to changes in the downstream ORF. The HL4, HL2 and HL1 libraries previously tested with yEGFP are shown here with the mRuby2 ORF. The unmodified GAL1-based promoter is shown as a reference in the top rown. ⟨MFE⟩ indicates the average MFE of the corresponding library. (**B**) Robustness of the library predictions to the use of different promoters upstream of the 5'UTR hairpin. The HC1 library consisting of hairpins with an average MFE of -28.9 kcal/mol in the 5'UTR for 5 different constitutive promoters of decreasing strength. For comparison, the promoters with a control 5'UTR with no structure are shown in the first column.

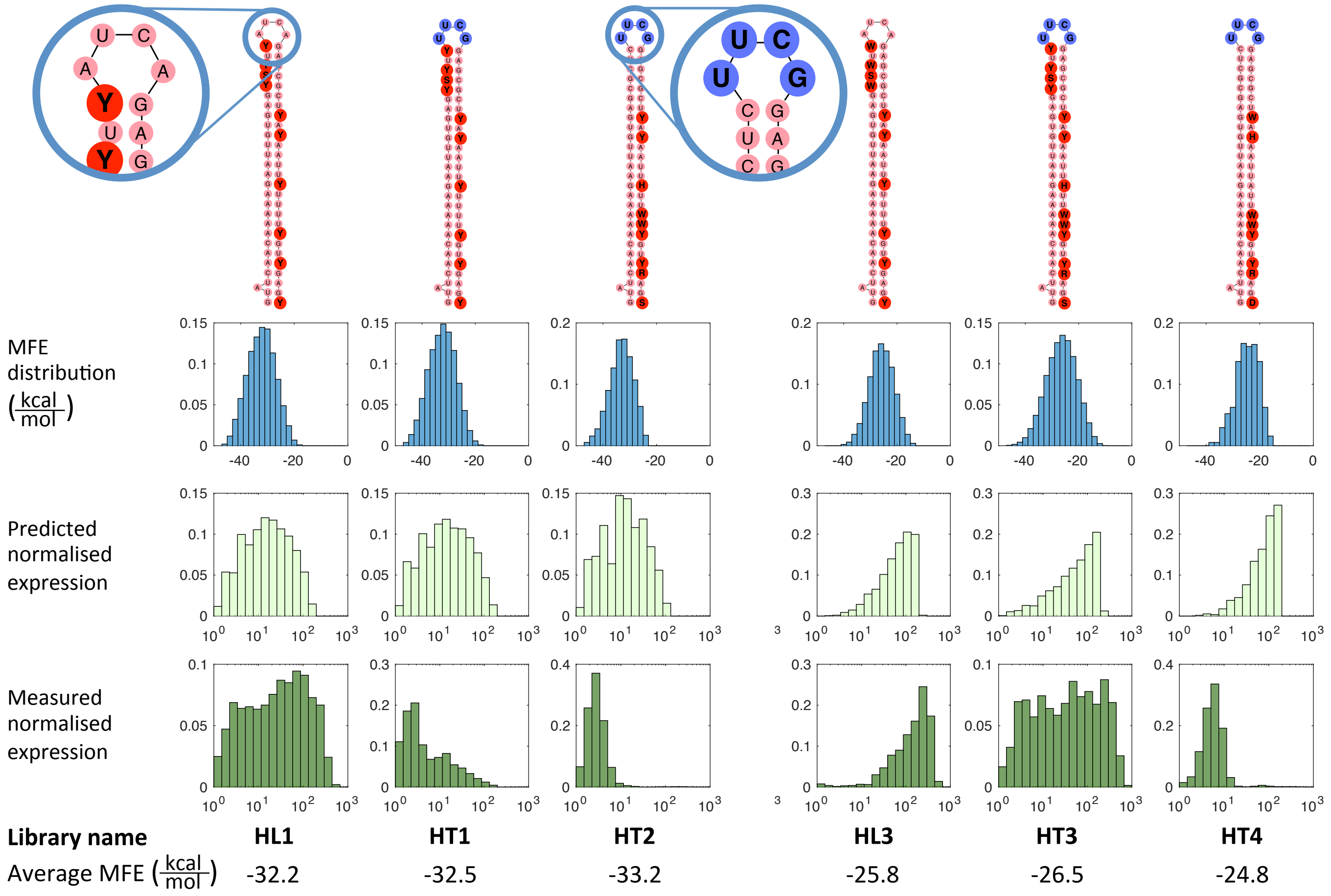

Figure S5: Two sets of libraries characterising the effect of the highly stable tetraloop on fluorescence levels. Left set (first three columns) each have MFE averages of -32 to -33 kcal/mol. The right set has MFE averages of -24.5 to -26.5 kcal/mol, as indicated below the histograms. The illustrations at the top show the scaffold hairpin and introduced degeneracies for each library. The blue bases in the RNA structures indicate the presence of the strong UUCG tetraloop. The top row of normalised histograms shows the MFE distributions for the 5'UTRs of each of the 6 libraries. The horizontal axes for these panels ranges on a linear scale from -50 kcal/mol on the left to 0 kcal/mol on the right. The middle row converts these into a distribution of predicted expression levels. Expression profiles are predicted by mapping the structure MFE to an associated expression level using the function derived in **Figure 2A and B** of the main manuscript. The third row shows the distribution of fluorescence levels in yeast cells as measured by flow cytometry. In the lower two rows, the horizontal axis corresponds to normalised green fluorescence (a unit-less quantity) ranging on a logarithmic scale from 1 on the left to 1000 on the right. Note that measured expression levels deviate substantially from the predictions for libraries that contain a tetraloop. Note also that libraries HL1 and HT1 are identical apart from the loop sequence, that libraries HT2 and HT4 have no degeneracies in the section of the stem that is directly adjacent to the tetraloop sequence and the dramatic effect these properties have on the measured expression.

References

[1] M. E. Lee, W. C. DeLoache, B. Cervantes, and J. E. Dueber, "A highly characterized yeast toolkit for modular, multipart assembly," *ACS Synth Biol*, vol. 4, pp. 975–86, Sep 2015.

[2] R. D. Gietz and R. H. Schiestl, "High-efficiency yeast transformation using the liac/ss carrier dna/peg method," *Nature Protocols*, vol. 2, no. 1, pp. 31–34, 2007.

[3] C. R. Woese, S. Winker, and R. R. Gutell, "Architecture of ribosomal rna: constraints on the sequence of "tetra-loops"," *PNAS*, vol. 87, pp. 8467–71, Nov 1990.

[4] C. C. Correll and K. Swinger, "Common and distinctive features of gnra tetraloops based on a guaa tetraloop structure at 1.4 a resolution," *RNA*, vol. 9, pp. 355–63, Mar 2003.

[5] M. Molinaro and I. Tinoco, Jr, "Use of ultra stable uncg tetraloop hairpins to fold rna structures: thermodynamic and spectroscopic applications," *Nucleic Acids Res*, vol. 23, pp. 3056–63, Aug 1995.

[6] F. M. Jucker and A. Pardi, "Solution structure of the cuug hairpin loop: a novel rna tetraloop motif," *Biochemistry*, vol. 34, pp. 14416–27, Nov 1995.

[7] Q. Zhao, H.-C. Huang, U. Nagaswamy, Y. Xia, X. Gao, and G. E. Fox, "Unac tetraloops: to what extent do they mimic gnra tetraloops?," *Biopolymers*, vol. 97, pp. 617–28, Aug 2012.

[8] H.-C. Huang, U. Nagaswamy, and G. E. Fox, "The application of cluster analysis in the intercomparison of loop structures in rna," *RNA*, vol. 11, pp. 412–23, Apr 2005.

[9] R. M. Dirks, M. Lin, E. Winfree, and N. A. Pierce, "Paradigms for computational nucleic acid design," *Nucleic Acids Res*, vol. 32, no. 4, pp. 1392–403, 2004.

[10] J. A. Garcia-Martin, P. Clote, and I. Dotu, "Rnaifold: a web server for rna inverse folding and molecular design," *Nucleic Acids Res*, vol. 41, pp. W465–70, Jul 2013.

[11] R. Lorenz, S. H. Bernhart, C. Höner Zu Siederdissen, H. Tafer, C. Flamm, P. F. Stadler, and I. L. Hofacker, "Viennarna package 2.0," *Algorithms Mol Biol*, vol. 6, p. 26, 2011.

[12] S. Dvir, L. Velten, E. Sharon, D. Zeevi, L. B. Carey, A. Weinberger, and E. Segal, "Deciphering the rules by which 5'-utr sequences affect protein expression in yeast," *PNAS*, vol. 110, pp. E2792–801, Jul 2013.

[13] K. J. Livak and T. D. Schmittgen, "Analysis of relative gene expression data using real-time quantitative pcr and the 2(-delta delta c(t)) method," *Methods*, vol. 25, pp. 402–8, Dec 2001.
